# Transoral robotic surgery in head and neck cancer: an umbrella review of meta-analyses

**DOI:** 10.1097/JS9.0000000000004717

**Published:** 2026-03-25

**Authors:** Chuan-Lu Shen, Zi-Yue Fu, Yan-Qiang Zhang, Shu Wang, Si-Yue Yin, Yun-Xia Ma, Yi-Wei Liu, Liang Zhang, Shan-Wen Chen, Yan-Xun Han, Yu-Chen Liu, Kai-Le Wu

**Affiliations:** a Department of Otolaryngology, Head and Neck Surgery, The First Affiliated Hospital of Anhui Medical University, Hefei, Anhui, China; b Department of Oncology, The First Affiliated Hospital of Anhui Medical University, Hefei, Anhui, China

**Keywords:** head and neck carcinoma, transoral robotic surgery, umbrella review

## Abstract

**Background:**

The aim of this umbrella review is to investigate the advantages of primary transoral robotic surgery (TORS) over other treatments such as open surgery (OS), transoral laser microsurgery (TLM) and primary radiotherapy in the diagnosis management of head and neck cancer (HNC).

**Materials and methods:**

A comprehensive search for meta-analyses of comparisons between TORS of HNC and other treatments such as OS, TLM and primary radiotherapy was conducted in the PubMed, Embase, and Cochrane library databases. The primary outcomes assessed include oncological outcomes, surgical outcomes, function outcomes and complicates. The AMSTAR-2 tool was used to assess the quality, which consists of 16 entries, with final quality outcomes categorized as “critically low quality,” “low quality,” “moderate quality,” and “high quality.”

**Results:**

Of the 1424 articles retrieved, 14 studies were eventually included for the comprehensive umbrella meta-analysis. TORS was superior to OS in terms of survival rate, length of hospital stay, cosmetic outcome, quality of life, surgical efficacy, and recognition of HNC with unknown primary lesion, but no significant difference was seen between them and TLM or radiation therapy.

**Conclusion:**

There are few studies on head and neck tumors other than oropharyngeal cancer, and there is a lack of studies on specific tumor stages or HPV status of the population, which does not allow for the provision of personalized treatment recommendations based on the patient’s own situation, and there is a need to carry out more randomized controlled trials on the specific type and status of the patient in future.

## Introduction

Head and neck cancer (HNC) is a group of malignant tumors arising from the oral cavity, hypopharynx, nasopharynx, oropharynx, lips, nasal cavity, sinuses and salivary glands. The incidence has increased over the past decades, and HNC is the eighth most common cancer in the world by 2020^[1,2]^. Treatment is a serious challenge for HNC since its anatomical complexity and structural sensitivity. Traditional treatment approaches rely on open surgery (OS) paired with postoperative radiotherapy, which typically necessitates large incisions to access the oropharynx, creates obvious scars, and impairs the patient’s swallowing and speech functions. Despite significant improvements in local control rates, functional impairment and lower quality of life have a negative impact on treatment outcomes.

**HIGHLIGHTS**

- First umbrella meta-analysis in robotic surgery for head and neck cancer.
- Aim to investigate the advantages of primary TORS over other treatments.
- Lack of studies on specific tumor stages or HPV status of the population.

Minimally invasive surgery has grown in popularity as technology has advanced. In 1993, Steiner found transoral laser microsurgery (TLM) have much better functional outcomes than OS for laryngeal cancer^[3–5]^. However, the visualization and manipulation capabilities may be limited because of the intricate anatomy of the head and neck. Transoral robotic surgery (TORS) was rapidly developed to address these issues. TORS was first used in otorhinolaryngology by Weinstein and O’Malley in 2005^[6]^. US Food and Drug Administration (FDA) authorized the da Vinci Surgical System in 2009 for the removal of the T1–T2 malignant tumors of the oral cavity, pharynx, and larynx, as well as benign lesions^[7]^. Surgeons can complete the surgery with greater efficiency and accuracy using this technique due to its improved field of view and flexibility. According to data, compared to non-robotic surgery, TORS has a longer survival rate, a smaller margin positivity, and requires less adjuvant radiation^[8]^.

The incidence of squamous cell carcinoma (SCC) of the larynx and hypopharynx is decreasing in developed countries due to the reduction of tobacco and alcohol use^[9]^. In contrast, human papillomavirus (HPV)-associated SCC of the oropharynx has continued to rise, with data indicating that the number of patients diagnosed with HPV-positive oropharyngeal SCC increased from 16.3% in the 1980s to 71.7% in the 2000s^[10]^. At the same time, HPV-positive oropharynx SCC patients are usually younger and healthier than HPV-negative patients^[10–12]^, with a higher likelihood of survival, making the choice of optimal treatment especially crucial. Simultaneously, robotic surgery has advanced quickly to offer patients with early-stage tumors less invasive and better function-preserving treatment options. However, its disadvantages include being significantly slower than traditional surgery, being more costly, that’s restricts its use, and having no haptic feedback, which can lead to certain postoperative complications^[13]^. As the accuracy and safety of treatment have been further enhanced by advancements in radiation technology, patients continue to experience side effects such neurotoxicity, ototoxicity, nephrotoxicity, mucositis, neutropenia, and dysphagia^[14,15]^. High-level evidence-based research is needed to support the decision between conservative and surgical treatments, which will enable more patients to get individualized care.

The comparative effectiveness of treatments for HNC has been the subject of multiple meta-analyses. However, the quality of these studies varies, and the research population and outcome measures are comparatively uniform. In this study, we integrate all meta-analyses of TORS versus alternative treatments, including open surgery, TLM, and radiotherapy, for HNC in order to create a comprehensive umbrella meta-analysis of numerous interventions and outcomes. For the thorough evaluation of several meta-analyses or systematic reviews of robotic surgery for the treatment of HNC, this method is an advanced research methodology. Its strength is in resolving the shortcomings of conventional meta-analysis and incorporating greater levels of evidence. With its “synthesis of evidence” approach, umbrella meta-analysis effectively connects basic research and clinical practice in robotic surgery for HNC. Its goal was to identify and evaluate all available synthesized evidence, regardless of its quality, to provide a transparent and honest stocktake of the field. Moreover, our work has been reported in line with the TITAN criteria (Supplemental Digital Content Appendix 5, available at: http://links.lww.com/JS9/G596)^[16]^. We further declare that no artificial intelligence (AI) or AI-assisted technologies were utilized in the design, data analysis, or writing of this manuscript.

## Methods

### Protocol and registration

This paper is a pooled study of meta-analyses and systematic reviews to assess the similarities and differences in the clinical efficacy of TORS versus other treatments for HNC. The review strictly adheres to PRISMA (Preferred Reporting Items for Systematic Reviews and Meta-Analyses)^[17]^ and MOOSE (Meta-Analysis of Observational Studies in Epidemiology) guidelines (Supplemental Digital Content Appendix 1 and Appendix 2, available at: http://links.lww.com/JS9/G596)^[18]^. Furthermore, the study protocol was registered with PROSPERO (International Prospective Register of Systematic Reviews) .

### Eligibility criteria

Inclusion criteria were developed in strict accordance with PICOS criteria. Patients of any age with HNC were included; the intervention was TORS; comparative studies were other treatments such as TLM, OS and primary radiotherapy; and the primary outcome measures were surgical outcomes, oncological outcomes, functional outcomes and complication rates. The types of studies included were meta-analyses including single-group rate pooled analyses. In addition, (1) non-comparative studies, (2) no data analysis, (3) incomplete data or meta-analysis of only one original study, (4) non-English texts and animal studies, and (4) conference abstracts were excluded.

### Search strategy and study selection

A comprehensive search of PubMed, Embase, and the Cochrane library up to November 2024 for all relevant literature was performed. The specific search strategy is described in Supplemental Digital Content Appendix 3, available at: http://links.lww.com/JS9/G596. Initially, we screened the literature based on the title and abstract, followed by further screening by two independent authors based on the full text content, with any disagreements being discussed and consensus reached by the third author.

### Data extraction

Data extraction was performed independently by two authors, and any discrepancies were resolved by consulting a third author. We extracted author, year of publication, journal of publication, literature search source, type of study included, oncological outcomes (survival and mortality rates, recurrence rate, positive of surgical margins, number of lymph nodes), surgical outcomes (operation time, intraoperative blood loss, drainage volume and time, free flap reconstruction, tracheotomy), function outcomes(length of hospital stay, cosmesis satisfaction, quality of life), complications (post-operative bleeding, hematoma and seroma, chyle leakage and fistula, wound infection, nerve damage, respiratory damage), identification rates of carcinoma of unknown primary lesions, and overall effect estimates (relative risk, odds ratio, hazard ratio, cumulative effects, mean difference, weighted mean difference, and standardized mean difference with 95% confidence intervals and *P*-values). In addition, the type of effect model used in the meta-analysis (fixed or random effects), assessment of heterogeneity (*I*^2^ values), publication bias, GRADE quality assessment were extracted. Besides, the paper discusses whether potential sources of inter-study heterogeneity were explored through subgroup analyses or other methods.

### Methodological quality assessment

Two authors independently assessed the quality of the included literature using the AMSTAR-2 tool, and disagreements were discussed and agreed upon through consultation with a third author. The AMSTAR-2 consists of 16 entries that require authors to answer “yes” or “partial yes” or “no” or “no meta-analysis”, and the final quality results were categorized as “critically low quality,” “low quality,” “moderate quality,” and “high quality”^[19]^.

### Data analysis

Due to the inclusion of articles with duplicate studies and the extraction of more complex results with less detailed data, we directly described the results without doing a secondary meta-analysis. As an umbrella review, the primary objective was to systematically collate and appraise the findings of existing meta-analyses rather than to conduct a de novo meta-analysis of primary data. This approach was chosen to avoid the statistical pitfalls of pooling overlapping meta-analyses and to instead provide a high-level, critical overview of the consistency, quality, and gaps within the current body of synthesized evidence.

## Results

### Study characteristics

**Figure 1.**
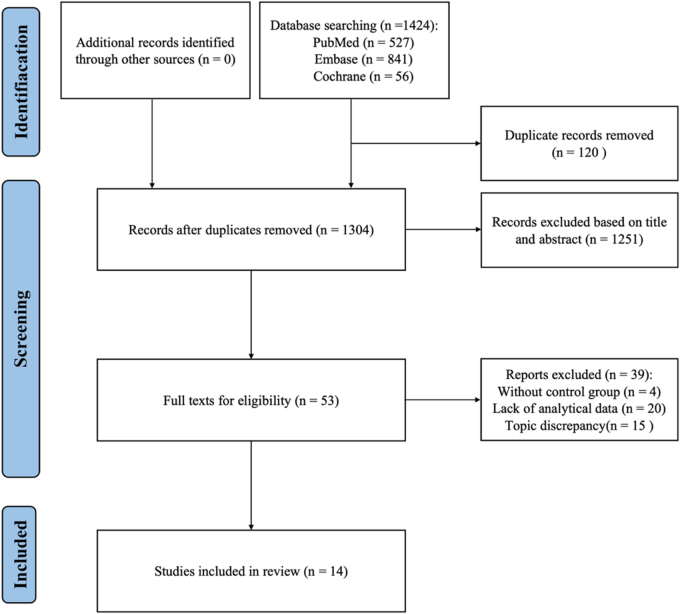
PRISMA flow diagram. Abbreviations: PRISMA, Preferred Reporting Items for Systematic Reviews and Meta-Analyses.

**Figure 2.**
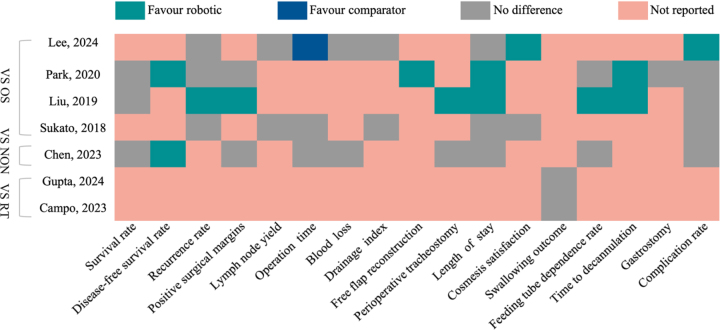
Overall findings of umbrella review. Abbreviations: OS, open surgery; NON, non-robotic surgery; RT, radiotherapy.

The PRISMA flow chart illustrates the selection process for TORS from initial search to inclusion in an overarching review (Fig. 1). From PubMed, Cochrane, and Embase, 527, 56, and 841 articles were retrieved, respectively; 120 articles were eliminated from duplicates, leaving 1304 articles; 1251 articles were eliminated based on abstracts and titles; 53 articles were included; and 39 articles were eliminated after their full texts were reviewed in accordance with the inclusion criteria; of these, 15 did not address the topic, 20 were merely systematic evaluations without meta-analysis data, and 4 lacked comparative studies. We finally included seven^[20–26]^ meta-analyses and seven single-arm pooled meta-analyses (Table 1)^[27–33]^. All findings can be seen in Figure 2. The remaining seven TORS versus non-robotic single-arm studies included four efficacy studies and three studies of aliasing in HNC of unknown primary lesion. Using AMSTAR-2 to assess the quality of the included literature, we found that only Liu’s study was considered “moderate,” while the remaining studies were of low quality. Specific evaluation results are shown in Supplemental Digital Content Appendix 4, available at: http://links.lww.com/JS9/G596. Most of the studies did not explore the risk of bias and did not explain its possible impact on the results. Detailed data of all included meta-analyses and single-arm pooled analyses are shown in Tables 2 and 3.

**Table 1:** Characteristics of the included studies.

First author, year	Journal title	Last search date	Databases searched	No. of studies included (T/RCT/PS/RS/CS/CSS)	Comparison	Tumor type or site	Interested Outcome	Grade results
Lee, 2024	Oral Oncology	August 2024	PubMed, Embase, Cochrane Library databases	8/0/2/6/0/0	RND vs CND	OC, NP, OP, HP, SG, LA	c, f, g, h, k, o, p, q, s	Not used
Gupta, 2024	European Journal of Surgical Oncology	January 2024	Embase, MEDLINE, CINAHL, Cochrane Library databases	6/2/2/1/0/0/1Secondary analysis of registry data	TORS vs RT	Tonsil, BoT, PW, SP, OP	j, l	Not used
Campo, 2023	Radiotherapy and Oncology	August 2022	MEDLINE, Embase, Cochrane Library databases	5/2/2/0/0/0/1Secondary analysis of registry data	TORS vs RT	Tonsil, BoT, SP, OP	j	Not used
Chen, 2023	Journal of Robotic Surgery	January 2023	PubMed, Web of Science, MEDLINE, Embase, Cochrane Library databases	7/0/1/6/0/0	TORS vs NON-RS	oropharyngeal squamous cell carcinoma	a, b, e, h, m, o, p, r, s	Not used
Park, 2020	European Journal of Surgical Oncology	July 2018	Ovid-MEDLINE, Ovid-Embase, Cochrane Library databases	9/0/3/6/0/0	TORS vs OS	OC, OP, SG, LA, PA	b, c, d, e, h, i, m, s	Not used
Liu, 2019	British Journal of Oral and Maxillofacial Surgery	July 2018	MEDLINE, Embase, CENTRAL, CNKI, CBM	13/1/0/0/9/3	TORS vs OS	OP recurrent, OP, BoT, AT, UAGT, HP, Supraglottis	a, c, e, h, l, j, m, n, r, s	Not used
Sukato, 2018	The Laryngoscope	NR	PubMed, Web of Science, EMBASE	6/0/4/2/0/0	RND vs CND	Head and neck squamous cell carcinoma	c, f, g, h, k, o, q, s	Not used
Virgilio, 2020^1^*	European Archives of Oto-Rhino-Laryngology	February 2020	PubMed, MEDLINE, Google Scholar, Cochrane Library databases	47/3/17/27/0/0	TORS/IMRT	Oropharyngeal squamous cell carcinoma	a, b, m, r	Not used
Asik, 2019*	The Journal of Laryngology & Otology	August 2017	PubMed, Sage, MEDLINE, Cochrane Library	24/NR	TORS/TLM	Supraglottic cancer	a, b	Not used
Virgilio, 2019^2^*	Journal of Clinical Medicine	August 2018	PubMed, Embase, Scopus	15/P and R	TORS/TLM	Hypopharynx squamous cell carcinomas	a, d, j, l	Not used
Ibrahim, 2018*	Head & Neck	March 2016	PubMed	17/NR	TORS/TLM/DT	BOT, SP, PW, tonsil	e, n, r, s	Not used
Al-lami, 2022*	Oral Oncology	September 2020	Embase, MEDLINE, CINAHL	30/4 multi-institutional and 24 single institutional case series/ 2 case reports	TORS/TLM/TOEC	CUP in the head and neck	Identification rate	Not used
Farooq, 2019*	Oral Oncology	July 2018	MEDLINE, CINAHL and Embase	21/3 multi-institutional and 16 single institutional case series/ 2 case reports	TORS/TLM	CUP in the head and neck	Identification rate	Not used
Maio, 2018*	Head & Neck	April 2018	Medline, PubMed, Embase, and Scopus	14/ 1 multi-institutional and 10 single institutional case series/ 3 case reports	TORS/EUA	SCCUP in the head and neck	Identification rate	Not used

a, Overall survival; b, Disease-free survival rate; c, Recurrence rate d, Mortality rate; e, Positive surgical margins; f, Overall lymph node yield; g, Positive lymph node yield h, Length of hospital stay; i, Free flap reconstruction; j, Swallowing outcome; k, Cosmesis satisfaction; l, Quality of life scores m, Perioperative feeding tube; n, Gastrostomy; o, Operation time; p, Blood loss; q, Drainage index; r, Perioperative tracheostomy; s, Complication rate.

AT, Anterior tongue; BoT, base of tongue; CND, conventional neck dissection; CS, cohort studies; CSS, cross-sectional study; CUP, carcinoma of unknown primary; DT, direct transoral; EUA, examination under anesthesia; HP, hypopharynx; IMRT, intensity‑modulated radiotherapy; LA, Larynx; NON-RS, non-robotic surgery; NP, nasopharynx; NR, not reported; OC, oral cavity; OP, oropharynx; OS, open surgery; PA, Pharynx; PS, prospective study; PW, pharyngeal wall; RCT, randomized controlled trial; RND, robotic neck dissection; RS, retrospective study; RT, radiotherapy; SCCUP, Squamous cell carcinoma of unknown primary; SG, salivary gland; SP, Soft palate; T, total No. of studies; TLM, Transoral Laser Microsurgery; TOEC, transoral endoscopic electrocautery; TORS, transoral robotic surgery; UAGT, Upper aerodigestive tract.

^*^ Single-arm cumulative meta-analysis

**Table 2:** Outcomes of meta-analysis.

First author, year	No. of studies	No. of patients	TORS	Comparator	Effect value		Estimates [95% CI]		*P*-Value	Heterogeneity (*I*^2^, *P*)
Overall survival rate										
Chen, 2023 (vs NON-RS)	4	284	136	148	OR (M-H, Random)	1.59	0.50	5.07	0.43	53%, *P* = 0.10
Mortality rate										
Park, 2020 (vs OS)	4	199	100	99	RR (M-H, Fixed)	0.81	0.30	2.20	0.68	0%, NR
Liu, 2019 (vs OS)	4	218	122	96	RR (M-H, Random)	0.81	0.32	2.08	0.67	0%, *P* = 0.98
Disease-free survival rate										
Chen, 2023 (vs NON-RS)	3	315	156	159	OR (M-H, Fixed)	3.43	1.92	6.15	<0.0001	0%, *P* = 0.85
Park, 2020 (vs OS)	5	329	165	164	RR (M-H, Fixed)	1.13	1.03	1.24	0.01	0%, *P* = 0.66
Recurrence rate										
Lee, 2024 (vs CND)	5	295	126	169	RD (NR)	0.00	−0.05	0.05	NR	0%, NR
Sukato, 2018 (vs CND)	3	109	41	68	RD (M-H, Random)	−0.051	−0.130	0.028	NR	NR
Park, 2020 (vs OS)	8	444	191	253	RR (M-H, Fixed)	0.66	0.36	1.22	0.18	0%, NR
Liu, 2019 (vs OS)	4	292	157	135	RR (M-H, Random)	0.48	0.25	0.91	0.02	0%, *P* = 0.84
Positive surgical margins										
Chen, 2023^1^ (vs NON-TOS)	5	305	146	159	OR (M-H, Random)	0.94	0.27	3.32	0.92	58%, *P* = 0.05
Chen, 2023^2^ (vs OS)	2	258	129	129	OR (M-H, Random)	0.45	0.14	1.44	0.18	66%, *P* = 0.09
Park, 2020 (vs OS)	4	286	139	147	RR (M-H, Fixed)	0.85	0.47	1.54	0.60	0%, NR
Liu, 2019 (vs OS)	7	483	242	241	RR (M-H, Random)	0.54	0.34	0.86	0.01	0%, *P* = 0.55
Overall lymph node yield										
Lee, 2024 (vs CND)	8	427	172	255	MD (NR)	−1.09	−3.18	1.00	NR	3.66%, NR
Sukato, 2018 (vs CND)	5	162	55	107	SMD (M-H, Random)	0.149	−0.358	0.656	0.565	NR
Positive lymph node yield										
Lee, 2024 (vs CND)	3	211	73	138	MD (NR)	−0.61	−2.20	0.98	NR	80.91%, NR
Sukato, 2018 (vs CND)	2	97	31	66	SMD (M-H, Random)	−0.026	−0.457	0.405	0.905	NR
Operation time										
Chen, 2023 (vs NON-RS)	2	97	51	46	MD (M-H, Random)	26.05	− 5.45	57.56	0.11	77%, *P* = 0.04
Lee, 2024 (vs CND)	6	273	114	159	MD (NR)	69.11	37.92	100.30	NR	83.34%, NR
Sukato, 2018 (vs CND)	5	149	51	98	SMD (M-H, Random)	2.63	1.321	3.938	0.000	NR
Blood loss										
Chen, 2023 (vs NON-RS)	2	87	41	46	MD (M-H, Fixed)	−20.87	−66.84	25.11	0.37	0%, *P* = 0.87
Lee, 2024 (vs CND)	5	195	75	120	MD (NR)	15.35	−7.39	38.10	NR	0%
Drainage volume										
Lee, 2024 (vs CND)	6	273	114	159	MD (NR)	15.29	−45.22	75.79	NR	76.08%, NR
Sukato, 2018 (vs CND)	2	62	23	39	SMD (M-H, Random)	0.291	−0.227	0.809	0.271	NR
Drainage time										
Lee, 2024 (vs CND)	6	269	119	150	MD (NR)	0.49	−0.02	1.00	NR	11.17%, NR
Free flap reconstruction										
Park, 2020 (vs OS)	3	161	76	85	RR (M-H, Fixed)	0.33	0.12	0.88	0.03	6%, *P* = 0.34
Perioperative tracheostomy										
Chen, 2023 (vs NON-RS)	4	260	126	134	OR (M-H, Random)	0.37	0.07	1.98	0.25	80%, *P* = 0.002
Liu, 2019 (vs OS)	4	243	118	125	RR (M-H, Random)	0.24	0.16	0.35	<0.0001	0%, *P* = 0.56
Length of hospital stay										
Chen, 2023 (vs NON-RS)	3	130	68	62	MD (M-H, Fixed)	−0.49	−1.88	0.89	0.49	0%, *P* = 0.55
Park, 2020 (vs OS)	4	180	90	90	WMD (M-H, Fixed)	−2.63	−4.74	−0.51	0.01	79%, NR
Liu, 2019 (vs OS)	3	128	64	64	WMD (M-H, Random)	−7.40	−0.08	−4.72	< 0.0001	0%, *P* = 0.84
Lee, 2024 (vs CND)	6	273	114	159	MD (NR)	1.07	−0.06	2.20	NR	0%, NR
Sukato, 2018 (vs CND)	4	129	50	79	SMD (M-H, Random)	−0.147	−0.534	0.240	0.456	NR
Cosmesis satisfaction										
Lee, 2024 (vs CND)	2	166	58	108	MD (NR)	2.03	1.48	2.57	NR	0%, NR
Sukato, 2018 (vs CND)	2	79	30	49	SMD (M-H, Random)	−1.485	−3.077	0.107	0.068	NR
Swallowing outcome (MDADI)										
Gupta, 2024 (vs RT)	5	477	195	282	MD (M-H, Random)	−0.24	−3.70	3.23	0.89	23%, *P* = 0.27
Campo, 2023 (vs RT)	5	478	195	283	MD (M-H, Random)	0.23	−2.57	3.02	0.87	0%, *P* = 0.44
Quality of life scores (EORTC-QLQ-C30 scores)										
Gupta, 2024 (vs RT)	3	172	96	76	MD (M-H, Random)	4.55	−1.44	10.53	0.14	0%, *P* = 0.69
Feeding tube dependence rate										
Chen, 2023 (vs NON-RS)	2	182	88	94	OR (M-H, Fixed)	1.19	0.48	2.95	0.70	18%, *P* = 0.27
Park, 2020 (vs OS)	3	139	69	70	RR (M-H, Fixed)	0.30	0.05	1.83	0.19	0%, NR
Liu, 2019 (vs OS)	2	180	90	90	RR (M-H, Random)	0.57	0.41	0.81	0.001	57%, *P* = 0.13
Time to decannulation										
Park, 2020 (vs OS)	2	91	44	47	WMD (M-H, Fixed)	−6.71	−8.40	−5.03	<0.00001	78%, NR
Liu, 2019 (vs OS)	4	184	94	90	WMD (M-H, Random)	−9.75	−11.80	−7.70	< 0.0001	0%, *P* = 0.39
Gastrostomy										
Park, 2020 (vs OS)	4	158	NR	NR	RR (M-H, Random)	0.69	0.25	1.92	NR	NR
Complication rate										
Postoperative bleeding rate										
Chen, 2023 (vs NON-RS)	2	182	88	94	OR (M-H, Fixed)	1.19	0.48	2.95	0.70	18%, *P* = 0.27
Park, 2020 (vs OS)	1	34	17	17	RR (M-H, Fixed)	0.33	0.01	7.65	0.49	NR
Liu, 2019 (vs OS)	2	184	NR	NR	RR (M-H, Random)	0.96	0.38	2.39	NR	NR
Lee, 2024 (vs CND)	7	339	123	216	RD (NR)	0.01	−0.04	0.07	NR	0%, NR
Hematoma										
Park, 2020 (vs OS)	3	196	69	127	RR (M-H, Fixed)	1.80	0.54	5.99	0.34	0%, NR
Liu, 2019 (vs OS)	1	47	NR	NR	RR (M-H, Random)	1.61	0.11	24.18	NR	NR
Sukato, 2018 (vs CND)	4	123	41	82	RD (M-H, Random)	0.019	−0.080	0.117	0.709	NR
Seroma										
Park, 2020 (vs OS)	3	196	69	127	RR (M-H, Fixed)	0.89	0.48	1.67	0.72	0%, NR
Lee, 2024 (vs CND)	5	262	98	164	RD (NR)	0.03	−0.06	0.12	NR	0%, NR
Sukato, 2018 (vs CND)	3	114	38	76	RD (M-H, Random)	−0.002	−0.120	0.116	0.974	NR
Chyle leak										
Park, 2020 (vs OS)	3	196	69	127	RR (M-H, Fixed)	0.81	0.24	2.74	0.73	0%, NR
Liu, 2019 (vs OS)	1	47	NR	NR	RR (M-H, Random)	1.61	0.11	24.18	NR	NR
Lee, 2024 (vs CND)	4	221	78	143	RD (NR)	0.01	−0.05	0.06	NR	0%, NR
Sukato, 2018 (vs CND)	2	84	27	57	RD (M-H, Random)	−0.011	−0.159	0.137	0.884	NR
Fistula										
Park, 2020 (vs OS)	3	218	111	107	RR (M-H, Fixed)	0.24	0.05	1.10	0.07	0%, NR
Liu, 2019 (vs OS)	4	265	NR	NR	RR (M-H, Random)	0.18	0.03	1.03	NR	NR
Wound infection										
Park, 2020 (vs OS)	2	86	41	45	RR (M-H, Fixed)	1.33	0.22	7.99	0.75	0%, NR
Liu, 2019 (vs OS)	4	230	NR	NR	RR (M-H, Random)	0.50	0.24	1.05	NR	NR
Lee, 2024 (vs CND)	3	64	23	41	RD (NR)	0.08	−0.06	0.23	NR	0%, NR
Sukato, 2018 (vs CND)	2	39	14	25	RD (M-H, Random)	0.000	−0.125	0.125	1.000	NR
Marginal nerve weakness										
Park, 2020 (vs OS)	2	143	49	94	RR (M-H, Fixed)	4.68	0.96	22.82	0.06	0%, NR
Lee, 2024 (vs CND)	4	285	110	175	RD (NR)	0.08	0.01	0.15	NR	0%, NR
Sukato, 2018 (vs CND)	4	118	44	74	RD (M-H, Random)	0.066	−0.055	0.186	0.284	NR
Postoperative spinal accessory nerve injury										
Lee, 2024 (vs CND)	5	227	92	135	RD (NR)	−0.01	−0.12	0.09	NR	55.13%, NR
Aspiration pneumonia										
Park, 2020 (vs OS)	1	34	17	17	RR (M-H, Fixed)	0.20	0.01	3.88	0.29	NR
Laryngeal stenosis										
Park, 2020 (vs OS)	1	34	17	17	RR (M-H, Fixed)	3.00	0.13	68.84	0.49	NR
Oedema of the airway										
Liu, 2019 (vs OS)	3	209	NR	NR	RR (M-H, Random)	0.90	0.46	1.76	NR	NR

CI, confidence interval; CND, conventional neck dissection; EORTC-QLQ-C30 scores, The European Organisation for Research and Treatment of Cancer Quality of Life Questionnaire Core 30; Fixed, fixed effect model; MDADI, M.D. Anderson Dysphagia Inventory; M-H, Mantel-Haenszel method; NON-RS, non-robotic surgery; NON-TOS, non-transoral robotic surgery; NR, not reported; NR, not reported; OR, odds ratio; OS, open surgery; Random, random effect model; RD, risk difference; RR, risk ratio; RT, radiotherapy; SMD, standardized mean difference; TORS, transoral robotic surgery; WMD, weighted mean difference.

**Table 3:** Outcomes of single-arm meta-analysis.

First author, year		No. of studies	No. of patients	Cumulative effects	Estimates [95% CI]		Heterogeneity (*I*^2^, *P*)
Overall survival						
Virgilio, 2020	TORS	15	NR	91.3%	81.2%	97.8%	84%, *P* < 0.001
	IMRT	25	NR	83.6%	76.9%	89.3%	87%, *P* < 0.001
Asik, 2019	TORS	5	56	82.5%	70.6%	91.0%	NR, *P* = 0.3279
	TLM	21	944	77.0%	70.6%	82.8%	NR, *P* < 0.0001
Virgilio, 2019	TORS	3	83	85.5%	55.8%	96.5%	77.7%, *P* = 0.011
	TLM	9	289	58.5%	46.6%	69.6%	79.3%, *P* < 0.0001
Disease-free survival							
Virgilio, 2020	TORS	11	NR	89.4%	82.7%	94.5%	54%, *P* = 0.02
	IMRT	21	NR	79.6%	70.6%	87.3%	86%, *P* < 0.001
Asik, 2019	TORS	4	45	87.0%	74.3%	94.9%	NR, *P* = 0.3666
	TLM	14	723	75.9%	68.3%	82.7%	NR, *P* < 0.0001
Death rate						
Virgilio, 2019	TORS	3	36	26.3%	9.0%	56.4%	82.19%, *P* = 0.004
	TLM	10	190	28.5%	24.1%	33.3%	29.15%, *P* = 0.176
Positive surgical margins						
Ibrahim, 2018	TORS	9	404	8.1%	4.9%	13.0%	NR
	TLM	3	314	8.7%	6.1%	12.5%	NR
	DT	3	310	9.6%	3.5%	23.9%	NR
Tracheostomy dependence						
Virgilio, 2020	TORS	NR	578	0.2%	0%	1.1%	0%, NR
	IMRT	NR	1016	0.7%	0%	1.1%	60%, NR
Ibrahim, 2018	TORS	6	300	6.0%	1.9%	17.2%	NR
	TLM	4	427	5.9%	1.7%	18.6%	NR
	DT	2	293	7.4%	1.9%	24.4%	NR
Feeding tube dependence						
Virgilio, 2020	TORS	NR	737	1.3%	0%	4.9%	69%, NR
	IMRT	NR	1995	4.0%	1.1%	8.4%	69%, NR
Gastrostomy						
Ibrahim, 2018	TORS	8	365	4.9%	2.4%	9.7%	NR
	TLM	5	358	5.4%	1.6%	16.5%	NR
	DT	1	102	3.9%	1.5%	10.0%	NR
Larynx function preservation						
Virgilio, 2019	TORS	3	102	92.6%	85.7%	96.4	0%, *P* = 0.932
	TLM	9	446	94.7%	91.1%	96.9%	22.2%, *P* = 0.246
Bleeding at oropharynx						
Ibrahim, 2018	TORS	6	276	4.1%	2.1%	8.1%	NR
	TLM	4	427	6.9%	2.9%	15.7%	NR
	DT	1	191	0.5%	0.1%	3.6%	NR
Identification rate						
Al-lam, 2022	TORS	22	551	60%	49%	70%	59.35%, NR
	TLM	5	149	80%	0.58	10.01	51.79%, NR
	TOEC	2	12	41%	5%	76%	0%, NR
Farooqa, 2019	TORS	15	464	74%	68%	79%	41.65, *P* = 0.05
	TLM	3	92	91%	85%	98%	NR
Maio, 2018	TORS	NR	NR	42%	9%	83%	NR
	TLM	NR	NR	31%	24%	40%	NR

CI, confidence interval; DT, direct transoral; EUA, examination under anesthesia; IMRT, intensity‑modulated radiotherapy; NR, not reported; RT, radiotherapy; TLM, transoral laser microsurgery; TOEC, transoral endoscopic electrocautery; TORS, transoral robotic surgery.

### Oncological outcomes

#### Survival and mortality rates

##### Overall survival and mortality

Three papers have compared survival between TORS and open surgery, and both survival and mortality TORS is superior.

##### Disease-free survival rate

The results of two meta-studies showed that the Disease-free survival rate (DFS) rate of TORS was significantly better than that of non-robotic surgery, and the difference was statistically significant in both cases (*P* < 0.001), and the low heterogeneity of the results reinforced the persuasiveness of the results.

#### Recurrence rate

We found significantly lower recurrence rates in the TORS than in the OS in two comparative studies.

#### Positive of surgical margins

We found the rate of positive tumor margins was statistically significant lower in Liu’s^[25]^ study for TORS comparing OS.

#### Number of lymph nodes

Studies by Sukato^[26]^ and Lee^[21]^ showed no significant difference in total lymph node yield as well as in positive lymph node yield in the robotic versus OS.

### Surgical outcomes

#### Operation time

Three meta-analyses showed longer robotic procedures, with the two statistically different ones comparing robotic-assisted neck lymphatic dissection with conventional neck lymphatic procedures.

#### Intraoperative blood loss

We found that one study showed more blood loss in TORS than OS. The other showed less blood loss when compared with non-robotic surgery, with neither difference being statistically significant.

#### Drainage volume and time

There was no statistical difference in drainage volume and time between TORS and OS.

#### Free flap reconstruction

Park’s^[24]^ study showed the rate of free flap reconstruction was lower in TORS versus OS statistically.

#### Tracheotomy

Two surgeries showed that tracheotomy utilization was lower in the TORS and the difference was statistically significant in Liu’s study^[25]^.

### Function outcomes

#### Length of hospital stay

Most of the results indicated that robotic surgery shortened the length of stay, with a statistically significant difference in Liu’s^[25]^ study on robotic versus OS; whereas Lee’s^[21]^ study indicated that OS was shorter than that in the TORS, but the difference was not statistically significant.

#### Cosmesis satisfaction

Two meta-comparative studies on neck lymphatic dissection referring to patient cosmetic score data have shown that patients are more satisfied with the incision of robotic surgery than with conventional surgery, with Lee’s^[21]^ study showing a statistically significant difference.

#### Quality of life

We included two meta-analyses that primarily examined patients’ postoperative swallowing function and found little difference. Additionally, there was no difference between robotic surgery and radiotherapy for quality of life in the Gupta *et al* study^[20]^. Three publications explored the feeding tube rates of patients during treatment and showed that robotic surgery had lower feeding tube rates, and the difference was statistically significant in Liu’s^[25]^ comparison of robotic versus OS; In addition, the time to placement of feeding tubes was reported to be much lower for TORS than OS, and the difference was statistically significant.

### Complications

#### Post-operative bleeding

We did not find a uniform conclusion in the included studies that there was no significant difference between the robotic and non-robotic groups in terms of postoperative bleeding.

#### Hematoma and seroma

The incidence of hematoma was higher in robotic surgery compared with open surgery, but the difference was not significant. The results of the three meta-studies on the incidence of postoperative seroma were not uniform, but none of the results were significantly different.

#### Chyle leakage and fistula

We only find the incidence of fistulae was lower in the TORS group than OS, but the statistical difference was not significant.

#### Wound infection

There was no significant difference in wound infection rates between TORS and OS.

#### Nerve damage

All three studies on the outcome of marginal nerve injury showed a higher rate of injury in TPRS than OS, and there was a significant difference in that outcome in Lee’s study^[21]^.

#### Respiratory damage

The incidence of postoperative aspiration pneumonia and laryngeal oedema in patients was reported in Park’s study^[24]^, with the former having a lower incidence in the TORS and the latter in OS, neither of which was significantly different from the other. The incidence of airway oedema in the Liu^[25]^ robotic group was slightly lower than that in the OS, and no statistically significant difference was seen either.

### Single-arm pooled data results

Seven single-arm pooled meta-analyses were included, four of which focused on efficacy studies. We discovered that the TORS group had better survival outcomes and positive margin pooling rates than the groups that underwent microsurgery and primary radiotherapy, but the results of all these studies were highly heterogeneous. Robotic surgery as an emerging technology, has a much lower sample size than radiotherapy and microsurgery, making the results were less convincing. In terms of surgical efficacy, Virgilio *et al*^[28]^ found that tracheotomy rates of 0.2 and 0.7% in the TORS and intensity-modulated radiotherapy (IMRT) groups, respectively, and data from Ibrahim’s study^[32]^ showed tracheotomy rates of 6.0, 5.9, and 7.4% in the TORS, TLM, and direct transoral surgery (DT) groups, respectively. In terms of postoperative quality of life and functional recovery, Virgilio *et al*^[28]^ also reported tube-feeding rates of 1.3 and 4.0% in the TORS group and the radiotherapy group, respectively. In addition, Virgilio *et al*^[29]^ in another study reported a cumulative preservation of laryngopharyngeal function of 92.6% for TORS and 94.7% for TLM.

A total of three single-arm meta-analyses examining the results of TORS in the identification rates of carcinoma of unknown primary lesions in HNC were included. Al-lam *et al*^[27]^ reported the pooled data of TORS, TLM, and transoral endoscopic electrocautery (TOEC) identification rates of 60, 80, and 41%, respectively; in addition, further reports on lingual tonsillar TORS, TLM, and TOEC identification rates of 45, 50, and 25% as well as TORS, TLM identification rate of 26 and 41% in palatal tonsillectomy. Farooqa *et al*^[31]^ investigated the identification effect of transoral lingual root mucosal resection, and the pooled percentage of TORS and TLM identification rate was 74 and 91%, respectively. Maio *et al*^[33]^. mainly explored the validity of palatal tonsillectomy for the detection of SCC of unknown primary in the HNC, and the included patients did not undergo any other surgical investigations, and the physical and imaging findings also needed to be negative, with a pooled proportion of 42 and 31% for TORS and examination under anesthesia (EUA), respectively.

## Discussion

This is the first meta-reanalysis comparing robotic surgery to alternative methods for treating HNC. In our included articles, the main type of disease studied was oropharyngeal cancer with a small number of glottic and hypopharyngeal carcinoma, and the control group consisted of mainly OS.

### Summary of main findings

We found that the TORS had better DFS and positive margin rates than the non-robotic surgery group including OS and TLM, and most of the included studies controlled for potential confounders including HPV status, age, smoking, TNM staging and adjuvant radiotherapy. Unfortunately, there are no meta-analyses comparing TORS versus radiotherapy regarding oncological outcomes, but from the included single-arm pooled analyses, Virgilio *et al*^[28]^ demonstrated that TORS was superior to the radiotherapy group in terms of overall survival (91.3 and 89.4%) and DFS (83.6 and 79.6%). For the study of surgical efficacy, we found that the operative time for robotic neck lymphatic dissection (ND) was significantly longer than that of OS, which may be related to the initial learning curve of TORS and the docking time of the robotic system^[34]^. However, there was no significant difference between the two in terms of postoperative drainage volume and drainage time for (ND), and the shorter hospital stay in the robotic group compared with OS, both of which suggest that the longer operative time may not have been substantially detrimental. In addition, Park *et al*^[24]^ explored the risk rate of free flap reconstruction showed a significantly higher success rate for TORS. Flap reconstruction is crucial for HNC surgery because most traumatic injuries can be closed directly or healed twice after surgery. However, if the defect is too big and affected by postoperative radiotherapy, it may result in serious complications. Barrette *et al*^[35]^ published a systematic review of reconstruction after TORS for HNC in 2022, with only 5 failures out of 260 free flap surgeries.

### Complications and technological considerations

The patient’s functional recovery following surgery is just as crucial as the survival result because of the head and neck region’s specialization. Our research revealed no discernible difference between TORS and radiation for swallowing function. One phase II randomized clinical trial of radiotherapy versus TORS for oropharyngeal SCC, ORATOR^[36]^, demonstrated that patients treated with radiotherapy had higher swallowing-related quality scores after 1 year of treatment, but this difference was not clinically significant.

Our investigation includes two studies by Sukato^[26]^ (2018) and Lee^[21]^ (2024) that compared robotic ND with conventional. Tumor control is a crucial factor in ND, and both results indicated that there was no discernible difference between these methods in terms of total lymph node yield, positive lymph node yield, or local recurrence rate, demonstrating that TORS is as effective than OS. Conventional ND usually leaves aesthetic problems^[37]^. A major advantage of robotic surgery is its minimally invasive approach, and we found that both studies showed that patients were more satisfied with the cosmetic outcome of TORS. Another advantage of TORS is the distance between the incision and the surgical bed. Because radiotherapy at a relatively new surgical incision is prone to many complications^[38]^, and chemoradiotherapy must frequently be postponed after surgery due to the possibility of complications. So the risk of complications is also greatly reduced when the incision is located away from the surgical bed and irradiated area^[39]^. In addition, Lee *et al*^[21]^ observed a higher rate of mandibular marginal nerve injury in the TORS in their 2024 study. This nerve injury could be attributed to surgical thermal damage or compression loss of the robotic arm.^[40]^ And due to the lack of haptic feedback in the da Vinci robotic system,^[41]^ it is highly likely that tissue damage or accidental injury could result from inappropriate force applied by the surgeon.^[42]^ A variety of haptic feedback systems are currently being investigated, and have been shown to reduce mean and maximal grip force, as well as enhance the detection of embedded blood vessels in soft tissues^[43]^. In addition, the Flex robotic system, which was developed specifically for TORS, has shown great potential. The Flex Robotic System represents a hybrid technology that integrates the flexibility of an endoscope to access the surgical site with the capacity to stiffen for the execution of the procedure. The flexibility of the device allows for 102° of freedom in angular motion, facilitating adaptation to the anatomical variability among patients and enabling access to less accessible anatomical areas^[44,45]^. So it is not only in terms of good field of view, but also in terms of haptic feedback, which is lacking in the da Vinci system, and which ensures the accurate identification of different tissues^[41,46]^. Despite the utilization of the Flex Robotic System for several years, it is imperative to incorporate long-term follow-up in subsequent studies to enhance comprehension of the efficacy of the procedure, particularly in the context of malignant diseases. To date, there have been no randomized controlled trials that have directly compared the Flex Robotic System with traditional surgery or other robotic systems. Consequently, it is imperative that future studies be conducted to provide more detailed clinical indications for the Flex Robotic System. However, little has been reported on the Flex robotic system, and further research is needed to help fully understand the similarities and differences between this technique and other surgical approaches. The integration of future technologies (such as artificial intelligence and augmented reality) with robotic platforms may have significant implications, representing a key direction for transforming the landscape of TORS clinical applications.

### Implications for HPV-positive disease and de-escalation

The number of HPV+ oropharyngeal cancer patients is increasing nowadays. However, the present regimen may be over-treatment of patients with HPV+ oropharyngeal cancer as it is more sensitive to radiotherapy, so a strategy of de-intensifying the treatment is proposed^[47]^. The aim is to maintain efficacy while reducing the burden of treatment-induced toxicity and not affecting the prognosis of these patients^[48]^. TORS with de-intensified adjuvant therapy is a promising alternative treatment option^[49]^. Only the ORATOR2 phase II randomized clinical trial^[50]^ has investigated the toxicities and survival of radiotherapy versus transoral surgery for HPV-related oropharyngeal SCC. In this trial of 61 patients with T1-T2 N0-2 HPV+ oropharyngeal SCC 27 patients underwent surgery of whom 25 underwent TORS and 2 underwent TLM. However, two treatment-related deaths occurred in the TORS group, a case of surgical site bleeding as well as a case of spinal discitis after receiving adjuvant RT, while the radiotherapy group had different toxicities including anorexia, dyspepsia, and higher long-term creatinine values, but quality scores of swallowing function were high for both treatment, and authors recommended caution with the use of TORS. But Virgilio^[51]^ suggested, in response to ORATOR2, that the two deaths are likely to be an incidental event with too small sample size or the result of adjuvant RT. Moreover, relevant large sample studies have confirmed the safety of TORS^[52,53]^. Therefore, TORS remains an appropriate treatment for early-stage oropharyngeal SCC, and further trials with uniform standards and larger sample sizes are needed.

In addition, we included three single-arm pooled analyses on identifying unknown primary foci of HNC. Although the use of TLM predates TORS, the number of included studies of TORS is more than that of TLM, suggesting that there may be a publication bias in the results of TLM identification rates over TORS. Al-lami *et al*^[27]^ analyzed in his study the HPV+ and HPV − patients with an overall identification rate of 82% (178/216) and 12% (7/59), respectively, but due to the large amount of missing data a pooled analysis was not performed as well as subgroup analyses of the TORS and TLM groups’ identification rates according to HPV status. The irradiation field and radiation-related side effects reduce greatly when radiotherapy is performed after the primary lesion are clearly identified. Patients treated for the primary lesion have a better prognosis and are easier to follow up with follow-up, and studies have demonstrated that HNC in which the primary lesion have been identified have a better long-term survival rate.

The high cost of TORS is primarily driven by the initial procurement of the robotic system, annual maintenance fees, and expensive single-use specialized instruments, which limits the widespread use of robotic surgery^[54]^. Despite high upfront costs, TORS may partially offset these expenses through significantly reduced hospital stays, decreased intraoperative blood loss and transfusion requirements, lower rates of tracheostomy and free flap reconstruction, and potentially reduced costs associated long-term swallowing dysfunction rehabilitation and care. We therefore conclude that the cost-effectiveness of TORS is highly dependent on surgical volume (center effect), the local healthcare payment system, device utilization rates, and whether it successfully avoids costly long-term complications. Future cost-effectiveness studies need to quantify these factors in more detail. Current research on the cost-effectiveness of TORS versus non robotic surgery cost analysis studies have produced conflicting results, but generally support TORS as a cost-effective alternative to radiotherapy^[55–57]^.

### Limitations and future directions

TORS, as a minimally invasive technique, offers potential advantages such as improved visualization, instrument articulation, and ergonomics for the surgeon when compared to traditional open surgery. However, as this review highlights, these technical features do not universally translate into superior clinical outcomes across all endpoints, and the technology carries specific limitations, including the lack of haptic feedback and significant cost. However, surgery cannot treat all HNC, especially as most current data only support the use of TORS in T1, T2, and some specific T3 tumors, and radiation therapy is essential for some local disease areas and critical site tumors. The most significant limitation of this umbrella review is that its conclusions are built upon a foundation of methodologically fragile meta-analyses. This inherently undermines the strength of any synthesized findings and dictates that they must be interpreted with utmost caution. This comprehensive review on TORS for HNC is subject to limitations, the most notable of which pertains to the quality of the included evidence. The AMSTAR-2 assessment identified that the majority of studies possessed “critically low” or “low” methodological quality. As the strength of any systematic review’s conclusions is contingent upon the rigor of its constituent studies, this finding directly compromises the certainty of our findings. Secondly, there were few randomized controlled trials, and most of the original studies were cohort studies or case series, increasing the risk of bias. Moreover, the inability to conduct subgroup analyses of patients’ HPV status and tumor staging due to insufficient data significantly undermines the clinical applicability of our research.

## Conclusion

TORS has some survival advantages over OS for early-stage (T1–T2) oropharyngeal cancer, but there is a lack of studies comparing robotics with radiation and TLM, and because TORS was initially applied to oropharyngeal cancer, the included studies were almost exclusively for oropharyngeal cancer, with a lack of studies of robotics for other types of cancers. While cohort studies and meta-analyses suggest TORS offers functional and oncologic benefits, the results of randomized trials like ORATOR and ORATOR2 introduce crucial caution. They demonstrate that the functional advantages of TORS over modern radiotherapy may be marginal at best, and reveal serious, albeit rare, safety concerns. It is known that HPV+ oropharyngeal cancer patients may have better survival outcomes, but there is still a lack of subgroup analyses according to HPV status, and it remains unanswered whether de-intensification can be implemented in HPV+ patients and how to develop a rational treatment plan. A large number of well-designed randomized controlled studies are needed to provide reliable reference information in the future. Treatment decisions should be based on multidisciplinary team assessment, comprehensive consideration of precise tumor stage and location, HPV status, patient comorbidities, and functional expectations. In centers with extensive robotic surgery experience, TORS is a recommended option for selected patients with T1–T2 oropharyngeal cancer with suitable anatomy.

## Data Availability

The original contributions presented in the study are included in the article/supplementary material. Further inquiries can be directed to the corresponding authors.

## Research registration unique identifying number (UIN)

International Prospective Register of Systematic Reviews (Registration No. CRD42024593094).
